# Ginsenoside PPD’s Antitumor Effect via Down-Regulation of mTOR Revealed by Super-Resolution Imaging

**DOI:** 10.3390/molecules22030486

**Published:** 2017-03-19

**Authors:** Bo Teng, Junguang Jiang, Lijing Zhao, Jing Gao, Junyu Chen, Zhe Liu, Hongda Wang, Binfeng Lu

**Affiliations:** 1 Department of Otolaryngology Head and Neck Surgery, The Second Hospital, Jilin University, Changchun 13041, Jilin, China; Jack.c.j.y@163.com (J.C.); keke_liu@foxmail.com (Z.L.); 2 State Key Laboratory of Electroanalytical Chemistry, Changchun Institute of Applied Chemistry, Chinese Academy of Sciences, Changchun 13021, Jilin, China; jiangjg@ciac.ac.cn (J.J.); gaojing@ciac.ac.cn (J.G.); hdwang@ciac.ac.cn (H.W.); 3 School of Nursing, Jilin University, Changchun 13021, Jilin, China; zhao_lj@jlu.edu.cn; 4 Department of Immunology, University of Pittsburgh, Pittsburgh, PA 15261, USA; binfeng@pitt.edu

**Keywords:** PPD, laryngeal cancer, mTOR, antitumor, dSTORM

## Abstract

Derived from *Panax ginseng*, the natural product 20(*S*)-Protopanaxadiol (PPD) has been reported for its cytotoxicity against several cancer cell lines. The molecular mechanism is, however, not well understood. Here we show that PPD significantly inhibits proliferation, induces apoptosis and causes G2/M cell cycle arrest in human laryngeal carcinoma cells (Hep-2 cells). PPD also decreases the levels of proteins related to cell proliferation. Moreover, PPD-induced apoptosis is characterized by a dose-dependent down-regulation of Bcl-2 expression and up-regulation of Bax, and is accompanied by the activation of Caspase-3 as well. Further molecular mechanism is revealed by direct stochastic optical reconstruction microscopy (dSTORM)—a novel high-precision localization microscopy which enables effective resolution down to the order of 10 nm. It shows the expression and spatial arrangement of mTOR and its downstream effectors, demonstrating that this ginsenoside exerts its excellent anticancer effects via down-regulation of mTOR signaling pathway in Hep-2 cells. Taken together, our findings elucidate that the antitumor effect of PPD is associated with its regulation of mTOR expression and distribution, which encourages further studies of PPD as a promising therapeutic agent against laryngeal carcinoma.

## 1. Introduction

Head and neck squamous cell carcinoma (HNSCC) is the sixth most common cancer worldwide [1]. Laryngeal cancer represents the largest known subgroup, with an estimated 156,877 new cases and 83,376 deaths worldwide annually according to GLOBOCAN 2012, and more importantly, the data is still increasing annually [2]. However, one of the continuing challenges in the therapeutic approach towards laryngeal squamous cell carcinoma (LSCC) is the development of resistance to chemotherapy and radiation treatment [3,4,5]. Although X-ray dose escalation techniques have been used to counteract radioresistance, dose escalations to 64.4 Gy in T1–T2 glottic carcinoma yield significant acute and late toxicities [6]. As a result, a significant number of researches have aimed at the identification of novel agents capable of overcoming these adaptations. Compared to chemical medicine, herbal medicine, with low side effect, might be a useful chemotherapeutic agent to consider for the future treatment of laryngeal carcinoma.

Ginseng, the root of different *Panax* species (*Araliaceae*), has been used in East Asian countries for at least 5000 years. It is well known for its medical properties, including anticancer, anti-inflammatory and neuroprotective activities [7,8]. The product, 20(*S*)-Protopanaxadiol (PPD)—a dammarane-type triterpenoid sapogenin isolated from both Asian ginseng (*Panax ginseng* C.A. Meyer) and American ginseng (*Panax quinquefolius* L.)—exhibits promising anticancer clinical applications due to its low toxicity to normal cells [7]. It has demonstrated excellent anticancer effects against multiple tumor types in vitro and in vivo [9,10] due to the down-regulation of multiple signaling pathways [9]. Among these signaling pathways implicated in cancers, it is worth noting that PI3K/Akt/mTOR is the most frequently altered pathway in human cancers and controls many important processes [11]. mTOR is a kind of Ser/Thr kinase that is a target member of rapamycin. It is one of the most important effecters in this pathway and characteristics for cancer development, such as cell cycle, cell survival, metabolism, motility, angiogenesis, chemoresistance and genomic instability [11]. Abundant studies have indicated that deregulation and aberrant activation of PI3K-α and its downstream targets are linked to tumorigenesis and tumor maintenance in a variety of cancers, including lung, breast, prostate, colon, and brain cancers [12,13,14]. Thus, this pathway is regarded as an attractive candidate for therapeutic interventions.

Our previous reported results have demonstrated that expression levels of mTOR and its effectors are associated with the pathological differentiation of laryngeal carcinoma tissues [15]. However, to the best of our knowledge, PPD has not been tested in laryngeal carcinoma Hep-2 cells and there are still many issues to be resolved: (1) the anticancer effect of PPD and its molecular mechanism in laryngeal carcinoma Hep-2 cells; (2) how PPD affects the localization and expression levels of signal molecules in mTOR pathways; (3) whether there is an intimate relationship between PPD anticancer function and the distribution features of mTOR and its downstream targets. 

To elucidate the topics above, common methods such as Western blotting, immunofluorescence and flow cytometry need to be applied to quantitatively analyze the ensemble level of many biomolecules. Besides, new methods capable of directly monitoring the distribution and organization of proteins with nanometer precision at the single molecule level are imperative as well. In the recent past, a number of ‘super-resolution’ strategies have been developed that allow the resolution limit of ~200 nm to be bypassed [16], for instance PALM [17], (f) PALM [18], STORM [19], Dstorm [20], STED [21], SSIM [22], etc. A certain embodiment of super-resolution microscopy used here is direct Stochastic Optical Reconstruction Microscopy (dSTORM), having the advantage of using commercial fluorophores that can be switched between a fluorescent on and a non-fluorescent off state. Only a few, sparsely distributed molecules are randomly photoactivated to their fluorescent on state, while others are in the off state. By repeated acquisition, the positions of individual molecules are identified to a precision that markedly exceeds the diffraction limit. Therefore, it greatly facilitates both overall and single molecule study of biological processes to combine the common biochemical methods with dSTORM [23,24,25].

In this work, by using many feasible methods including traditional biotechnologies and super-resolution fluorescence microscopy, we investigated the potential antitumor effect of ginsenside PPD in human laryngeal carcinoma Hep-2 cells and revealed that PPD’s antitumor effect was associated with mTOR signaling pathway. 

## 2. Results and Discussion

### 2.1. PPD Suppresses the Proliferation of Hep-2 Cells

**Figure 1:**
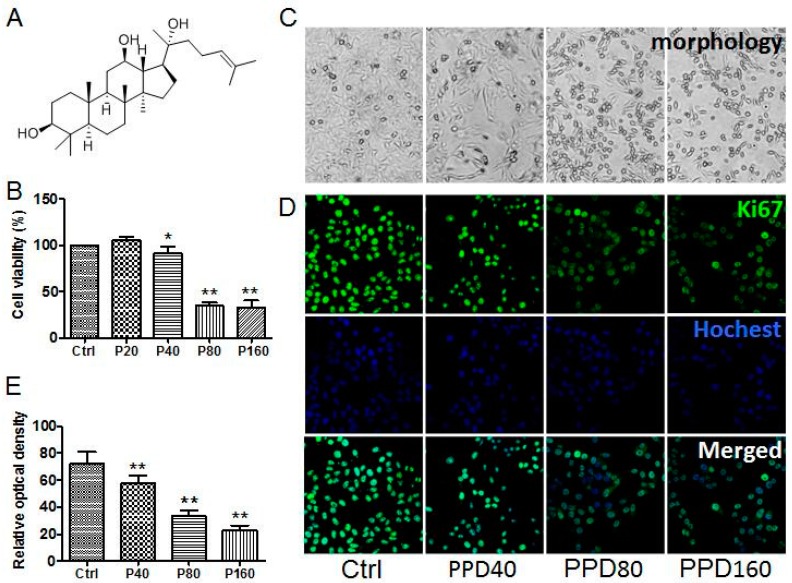
Inhibitory effect of 20(*S*)-Protopanaxadiol (PPD) on the proliferation of Hep-2 cells. (**A**) The chemical structure of PPD; (**B**) MTT assay measured the viability of Hep-2 cells treated with different concentrations of PPD for 24 h. Data are expressed as mean ± S.D*.*, * *p* < 0.05, ** *p* < 0.01, for PPD-treated cells vs. DMSO (Dimethyl Sulphoxide)-treated cells, *n* = 6; (**C**) Morphological images of Hep-2 cells in the presence of treatment as illustrated; (**D**) Confocal images of Ki67 and nucleus in Hep-2 cells with or without PPD treatment to detect cell proliferation. Ki67 was labeled with primary antibody and Alexa Fluor 488-conjugated goat anti-mouse IgG. Nuclei were stained with Hoechst; (**E**) The relative optical density of Ki67, which presents the ability of cell proliferation. Data are expressed as mean ± S.D., ** *p* < 0.01, compared to control, *n* = 3.

The structure of PPD is presented in Figure 1A. A previous work has studied 11 ginsenosides and reported that PPD is one of the most effective inhibitors of cancer cell growth and proliferation [26]. Since inhibiting cell growth is a critical action of anti-cancer drugs, we firstly carried out MTT assay to assess PPD potency and its effect on the viability of Hep-2 cells. As shown in Figure 1B, a high dose of PPD (≥40 μM) markedly inhibited the proliferation of Hep-2 cells, indicating that the inhibition of PPD was in a dose-dependent manner. Consistent with MTT results, we observed that the number of Hep-2 cells decreased gradually as the concentration of PPD increased (Figure 1C). Moreover, the reduction of Ki67 expression in PPD treated Hep-2 cells confirmed the inhibitory effect of PPD on cell proliferation as well (Figure 1D,E).

Of note, we found that Hep-2 cells grew moderately when the concentration of PPD was 20 μM, and the cell viability started to fall only when the concentration was over 40 μM (Figure 1B). It suggested that low dose of PPD may improve the survival rate of cells and protect cells, whereas high dose of PPD can suppress cell proliferation [27]. Some studies have also found that PPD plays a cytoprotective role through over-expressing Bip—a critical chaperone for Endoplasmic reticulum homeostasis—at low concentrations, while PPD significantly induces a proapoptotic protein CHOP at high concentrations [28]. Based on our observations, we conclude that PPD suppresses the proliferation of Hep-2 cells in a dose-dependent manner and determine an effective concentration threshold of PPD as an inhibitor of cancer cell growth, which is 40 μM. Accordingly, we applied the moderate concentrations of PPD (40 μM, 80 μM and 160 μM) in subsequent experiments.

### 2.2. PPD Induces G2/M Cell Cycle Arrest

**Figure 2:**
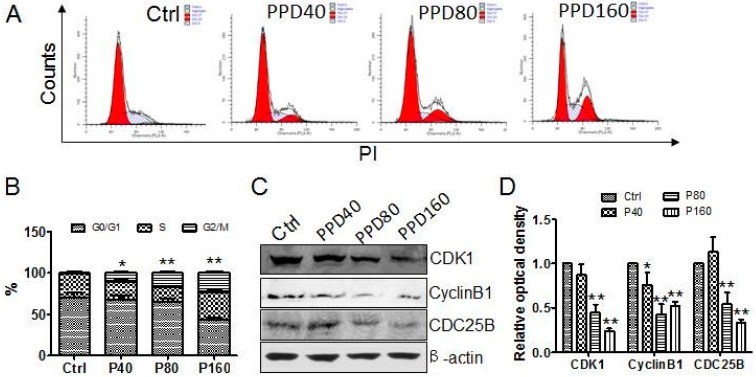
G2/M-phase arrest induced by PPD in Hep-2 cells. (**A**) FACS analysis of PPD inhibiting cell cycle in G2/M phase in Hep-2 cells. Cells were treated with 0, 40, 80 and 160 µM PPD for 24 h, respectively, and then fixed, labeled with PI (Propidium Iodide), and subjected to flow cytometry; (**B**) The percentage of cells in different phase as shown in (**A**). Data are expressed as mean ± S.D., * *p* < 0.05, ** *p* < 0.01, compared to control, *n* = 3; (**C**) Western blot analyses of the expression of cell cycle related proteins in Hep-2 cells treated with different concentration of PPD; (**D**) The quantization of the Western blot data after correction for the β-actin loading control. Data are expressed as mean ± S.D., * *p* < 0.05, ** *p* < 0.01, compared to control, *n* = 3.

As cell proliferation is achieved by cell division, we inferred that PPD inhibiting the proliferation of Hep-2 cells might be associated with its effect on the cell cycle. To ascertain this possibility, we analyzed cell cycle profiling of Hep-2 cells by flow cytometry after being treated with PPD. We observed that G0/G1 phase cells reduced significantly whereas G2/M phase cells kept an upward tendency after PPD treatment (Figure 2A,B). Moreover, the increase of the percentage of G2/M phase cells was proportional to PPD concentration. This finding indicated that PPD arrests Hep-2 cells in G2/M phase. Previous studies have shown that PPD induced cell cycle arrest in G0/G1 phase in A549 cells [10] or in G1 phase in LNCaP and PC3 cells [27]. Thus, all of the results validate the fact that PPD inhibits the cell cycle and that the phase that PPD arrests is related to the types of cancer cells.

Cyclin B is synthesized at G2 phase and accumulates in cytoplasm to drive cells from G2 phase to M phase. Cyclin B/CDK1 complex binds to the chromosome for the entry of prophase. CDC25B gradually increase and activated the Cyclin B/CDK1 by phosphorylation, and leads to replication of unstable microtubules and prophase entry. To investigate the potential molecular mechanisms of PPD to induce the G2 phase arrest, Western blot was employed to detect the expression of CDK1, Cyclin B1 and CDC25B. The results showed that the expression levels of these three kinds of proteins declined steadily along with the increasing concentration of PPD (Figure 2C,D). These data verify the ability of PPD to inhibit cell cycle in G2/M phase, which nicely explains why Hep-2 cells stop growing after PPD treatment and offers a possible mechanism for the antitumor effect of PPD.

### 2.3. PPD Promotes the Apoptosis of Hep-2 Cells

**Figure 3:**
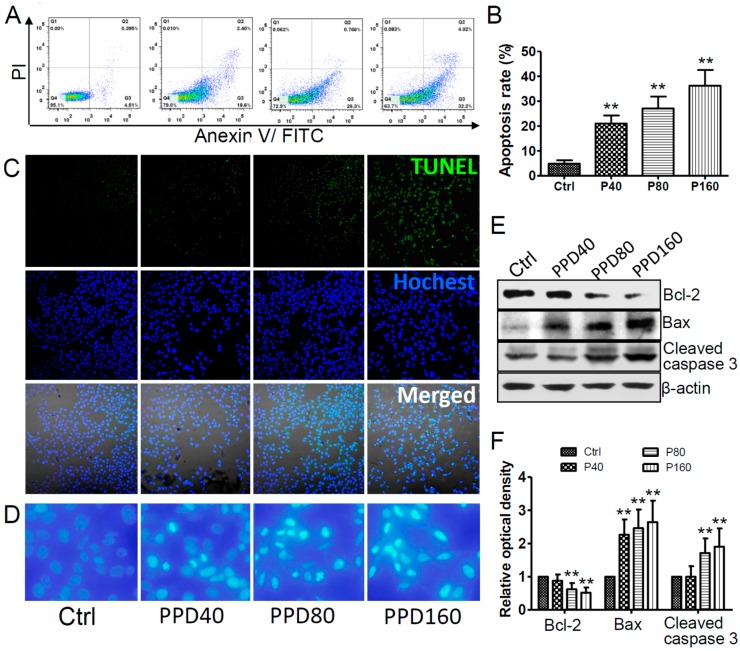
PPD induces the apoptosis of Hep-2 cells and affects the expression of apoptosis-related proteins. (**A**) Annexin V/PI analysis of the apoptosis of Hep-2 cells treated with different concentrations of PPD (0, 40, 80 and 160 μM). Viable cells with both negative Annexin V and PI are in Q4. Apoptotic cells with positive Annexin V and negative PI are in Q3. Secondary necrotic cells with both positive Annexin V and PI are in Q2. Mechanically injured cells with negative Annexin V and positive PI are in Q1; (**B**) Apoptosis rate of Hep-2 cells statistics from Annexin V/PI analysis. Data are expressed as mean ± S.D., ** *p* < 0.01, compared to control, *n* = 3; (**C**) TUNEL analysis of the apoptosis of Hep-2 cells treated with or without PPD; (**D**) Confocal images of nuclei stained with Hoechst 33342 in control and PPD treated Hep-2 cells; (**E**) Western blot analyses of the expression of various apoptosis-related proteins in Hep-2 cells with or without PPD treatment; (**F**) The quantization of the Western blot data after correction for the β-actin loading control. Data are expressed as mean ± S.D., ** *p* < 0.01, compared to control, *n* = 3.

Hep-2 cells showed a marked decline in cell number when treated with a high dose of PPD. Besides suppressing cell proliferation, another possible reason of the number decline may lie with PPD induced cell apoptosis. To test this possibility, we detected the apoptosis of PPD treated Hep-2 cells by Annexin V-FITC/PI staining and TUNEL assay. We found that both early and late apoptosis cells increased with the concentration of PPD (Figure 3A). The percentage of apoptotic cells treated with PPD was 21.2% ± 0.1%, 27.8% ± 2.8%, 38.1% ± 3.3%, respectively, whereas for DMSO-treated cells the percentage was 4.9% ± 0.1% (Figure 3B). We also detected apoptosis by TUNEL assay (Figure 3C), the results demonstrated that the percentage of TUNEL positive cells treated by PPD was 1.07% ± 0.1%, 17.7% ± 2.8%, 53.8% ± 3.3% and 64.8% ± 1.8%, respectively (data were not shown). The results of both measurements consistently demonstrated that PPD significantly induced Hep-2 cells apoptosis in a dose-dependent manner after exposed to various concentrations of PPD. These results were in agreement with previous studies that PPD exhibits a pro-apoptotic effect in multiple types of cancer cells, such as hepatocarcinoma, glioma, pancreatic cancer, breast cancer and prostate cancer [26,28]. Furthermore, from morphological analysis in cells stained with Hoechst 33342 (Figure 3D), the nuclei of cells were round and homogeneously stained in DMSO-treated cells. However, cells treated with PPD showed the significant condensation of nuclei, which is one of the typical morphological characterization of apoptotis [29]. Our data therefore verify the role of PPD in promoting the apoptosis of Hep-2 cells.

It is well known that cell apoptosis is a complex process controlled by multiple genes, in which a variety of apoptosis-related proteins are implicated. Next we investigated whether PPD can accelerate cell apoptosis through the regulation of apoptosis-related proteins. Thus, we chose several members of Caspase and Bcl-2 family as representatives to assess their expression levels after PPD treatment. Caspase-3 is considered to be the main terminal cleavage enzyme in cell apoptosis. It can be activated by both extrinsic (caspase-8) and intrinsic (caspase-9) pathways, which induces cell shrinkage, nuclear condensation and DNA fragmentation [30]. As shown in Figure 3E,F, the cleavage of caspase-3 increased gradually as PPD concentration went up, indicating that PPD promotes the activation of caspase-3 in a dose-dependent manner. In addition, the Bcl-2 family also has a great influence on cell apoptosis. It is comprised of anti-apoptotic proteins such as Bcl-2 and Bcl-xL, and pro-apoptotic proteins such as Bax, Bad, and Bak [31]. When the balance of the Bcl-2 family shifts to pro-apoptotic proteins, cytochrome C releases and cell apoptosis occurs. The results of Western blot showed that the expression level of Bcl-2 declined steadily along with the increasing concentration of PPD and the relative value in control cells was nearly two times of that in 160 μM PPD treated cells (Figure 3E,F). However, Bax expression had a significant rise and the relative value of 160 μM PPD treated cells was almost 250%. These data are consistent with those in a study of PPD treated human hepatocarcinoma HepG2 cells [28], demonstrating that PPD plays an important role in inhibiting anti-apoptotic proteins and promoting pro-apoptotic proteins. Taken together, these findings lend strong support to our hypothesis that PPD increases the function of inducing apoptosis by regulating apoptosis-related proteins in Hep-2 cells.

### 2.4. PPD Down-Regulates the Expression of mTOR Signaling Pathway

**Figure 4:**
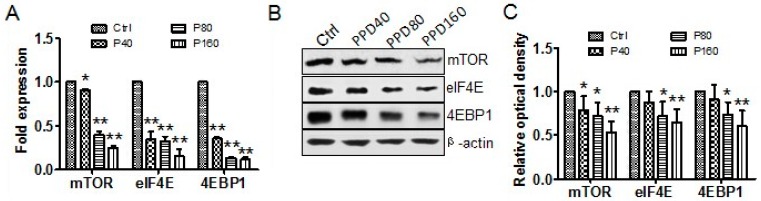
PPD affects the transcription and expression of mTOR and its downstream effectors. (**A**) Quantitative RT-PCR analyses of the effects of PPD on mTOR, 4EBP1 and eIF4E transcription. The relative levels of their mRNA are displayed in a histogram; (**B**) Western blot analyses of expression levels of mTOR, 4EBP1 and eIF4E in Hep-2 cells with different doses of PPD treatment; (**C**) The quantization of the Western blot data after correction for the β-actin loading control. Data in (**A**,**C**) are expressed as mean ± S.D., * *p* < 0.05, ** *p* < 0.01, compared to control, *n* = 3.

The antitumor effect of PPD against Hep-2 cells lies in inhibiting cell proliferation, blocking cell cycle and inducing cell apoptosis. A well-known signaling pathway, PI3K/AKT/mTOR, exerts a key role in these cellular processes [32] and has been found frequently over-activated in multiple types of cancers [33,34]. Activation of mTOR pathway leads to suppression of apoptosis and promotion of survival by phosphorylating the pro-apoptotic protein Bad of Bcl-2 family [35] and reducing the activity of caspase-3 [36]. In contrast, our results showed that the expression of pro-apoptotic protein Bax and cleaved Caspase-3 increased significantly after PPD treatment. Therefore, we supposed that PPD might play the efficient antitumor effect by regulating mTOR pathway. To test this idea, we analyzed the transcription and expression of mTOR and its downstream substrates, 4EBP1 and eIF4E, which represent best characterized targets of mTOR complex cascade [37]. As expected, the results of qRT-PCR analyses indicated the reduction of their mRNA caused by PPD treatment (Figure 4A). Western blot analyses also showed that PPD notably decreased the levels of mTOR and its downstream substrates in a dose-dependent manner (Figure 4B,C). Thus, these data demonstrate that PPD indeed modulates mTOR signaling pathway by down-regulating signal proteins of mTOR pathway, which may contribute to the antitumor effect of PPD.

**Figure 5:**
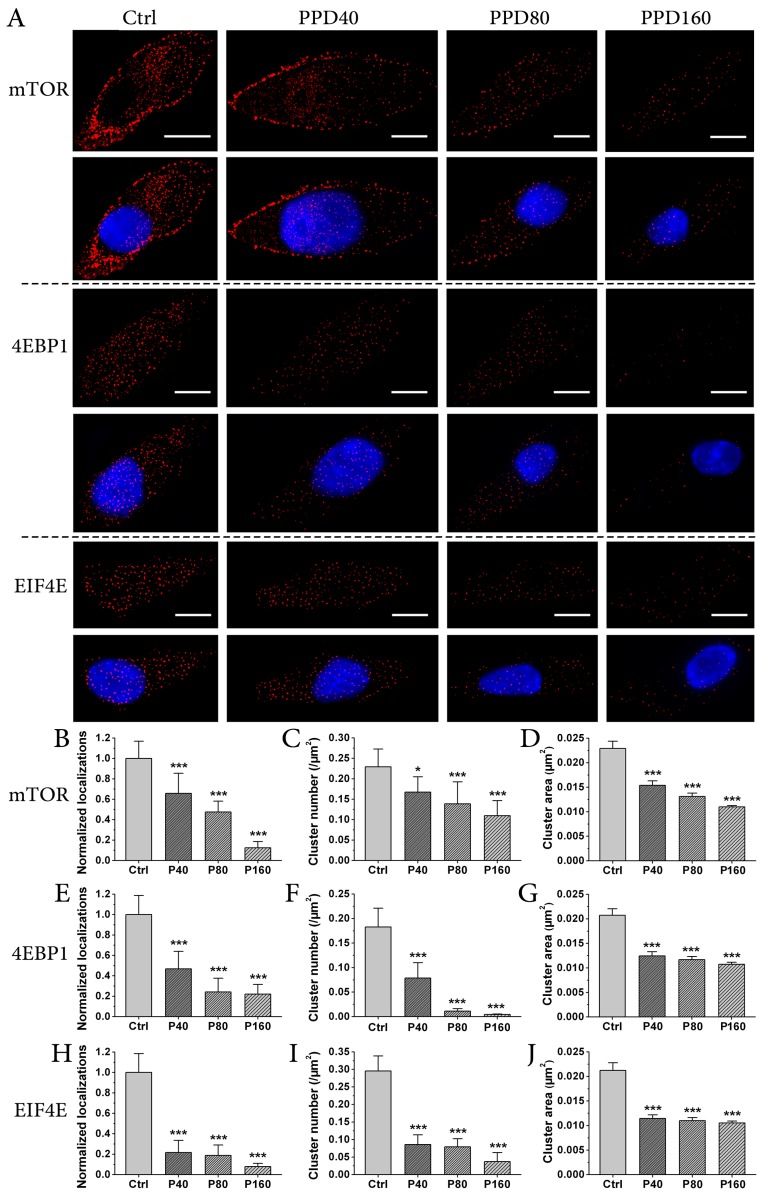
PPD regulates the distribution pattern of mTOR and its downstream effectors. (**A**) direct Stochastic Optical Reconstruction Microscopy (dSTORM) imaging of mTOR, 4EBP1 and eIF4E in Hep-2 cells treated with different doses of PPD. mTOR, 4EBP1 and eIF4E were labeled with individual primary antibodies and Alexa Fluor 647 secondary antibodies, nuclei were labeled with Hoechst 33342. Scale bars are 10 μm; (**B**,**E**,**H**) Normalized total localizations of reconstructed dSTORM images of mTOR (**B**), 4EBP1 (**E**) and eIF4E (**H**) in Hep-2 cells with different concentrations of PPD; (**C**,**F**,**I**) The cluster number of mTOR (**C**), 4EBP1 (**F**) and eIF4E (**I**) per μm^2^ in Hep-2 cells with or without PPD treatment; (**D**,**G**,**J**) The average cluster area of mTOR (**D**), 4EBP1 (**G**) and eIF4E (**J**) in Hep-2 cells with or without PPD treatment. Data in (**B**–**J**) are obtained from 20 cells in four independent experiments. Data are expressed as mean ± S.D., * *p* < 0.05, *** *p* < 0.001, compared to control, *n* = 10.

To detect the expression and distribution of mTOR and its downstream targets in detail, we used one of the super-resolution imaging techniques, dSTORM, to observe mTOR, 4EBP1 and eIF4E in Hep-2 cells treated with different concentrations of PPD or left untreated. As shown in Figure 5A, we found that these three kinds of proteins decreased after adding PPD. To accurately assess the effect of PPD on the expression levels of these three mTOR signaling constituents, we quantitatively analyzed normalized total localization number (Figure 5B,E,H) of each kind of protein. As the number of localizations for a single Alexa Fluor 647 secondary antibody is generally constant, the expression levels of labeled proteins can be estimated by the number of total localizations in their dSTORM images when the labeling ratio of target protein, primary antibody and secondary antibody is approximately 1:1:1. Using mTOR as an example, we measured the total number of localizations from a dSTORM image of mTOR in the control Hep-2 cell, and divided the number by the cell area. By calculating 20 cells in four independent experiments, we obtained the mean of localizations/area for control cells, and set this value as ‘1’. Other values for cells treated with different concentrations of PPD were calculated in the same way and normalized. The results showed that normalized localizations of these three kinds of proteins decreased significantly after PPD treatment and the reduced values were proportional to the concentration of PPD, indicating a sharp decline of the expression levels of mTOR and its downstream proteins by PPD treatment in a dose-dependent manner. These results were consistent with those from Western blot analyses (Figure 4), therefore further verifying an important role of PPD in weakening the expression of mTOR and its downstream effectors.

Interestingly, we also observed that these three kinds of proteins were mostly distributed in small clusters in control Hep-2 cells. Accordingly, we wondered whether PPD influenced on the distribution pattern of mTOR and downstream proteins. We performed image-based cluster analysis to compare the changes of their spatial distribution. By setting a threshold of the minimal cluster area (0.01 μm^2^) in Image J, clusters with the area ≥0.01 μm^2^ were extracted from dSTORM reconstructed images, and then parameters of cluster number per μm^2^ (Figure 5C,F,I) and cluster area (Figure 5D,G,J) of these three kinds of proteins in Hep-2 cells without or with PPD treatment were obtained. For cluster number, the value of mTOR decreased gradually as PPD concentration increased (Figure 5C). Thus, it is clear that PPD attenuates the formation of mTOR clusters. However, the values of 4EBP1 and eIF4E descended dramatically when PPD was only 40 μM, and even became less than 0.05 (0.0045 for 4EBP1, 0.037 for eIF4E) when PPD was 160 μM (Figure 5F,I). This finding suggest that the reduction of 4EBP1 and eIF4E clusters is not only due to the decrease of their upstream protein—mTOR—but is directly caused by PPD effect as well. For cluster area, the value of individual protein was a little more than 0.02 μm^2^ in control cells, and it fell to 0.01 μm^2^ when PPD was 160 μM. This result indicates that PPD induces the fragmentation of these three kind of clusters. Together, our data reveal the inhibition of PPD in cluster formation and cluster size of mTOR and its downstream proteins.

In recent years, much attention has been given to protein clustering. Numerous studies have provided evidence to elucidate the critical role of protein distribution and aggregation in promoting the interaction between these proteins and determining their proper function [23,38,39,40,41]. Here we find that mTOR, 4EBP1 and eIF4E also form clusters of 0.02 μm^2^ in Hep-2 cells. Because distances between proteins in clusters are short and their interactions are strengthened, it is possible that clustering distribution benefits fast signal transduction of mTOR pathway. However, PPD treatment reduces and disrupts clusters of these three kinds of proteins, which may be the way how PPD decreases the activity of mTOR signaling pathway. Based on above results, we conclude that PPD exerts an inhibiting effect on mTOR pathway by reducing both the expression levels and cluster formation of relevant signal proteins.

## 3. Materials and Methods

### 3.1. Cell Culture

Laryngeal carcinoma cell line Hep-2 were obtained from Cell Lines Bank, Shanghai Institute of Biological Sciences. Cells were maintained in RPMI-1640 medium (Gibco, Carlsbad, CA, USA) supplemented with 10% fetal bovine serum (Gibco) and antibiotics (100 IU/mL of penicillin and 100 mg/mL of streptomycin), and incubated at 37 °C in a humidified atmosphere of 95% air and 5% CO_2_.

### 3.2. Cytotoxicity Assay

Total saponins of *Panax ginseng* and 3% NaOH ethanol solution were dissolved and stirred in ethyl alcohol, left standing and filtered. The precipitate and the same amount of solid NaOH were dissolved in a solvent, then the mixture was heated at a high temperature for about 20 minutes. When the reaction was completed, we removed the reaction solution. The mixture was subjected to normal phase, reverse phase silica gel column chromatography [26], and purified to obtain the target compound. The molecular structure was elucidated based on spectroscopic data (IR, ^1^H-NMR, ^13^C-NMR, see Supplementary materials Figures S1–S3). The compound 20 (*S*)-PPD was then diluted in DMSO. The cytotoxicity of PPD was evaluated in Hep-2 cells by MTT assay. Briefly, cells were seeded into 96-well plates at 0.3 × 10^4^ cells/well and cultivated at 37 °C overnight, and then exposed to various concentrations of PPD (40 μM, 80 μM and 160 μM) for 24 h. Thereafter, 20 μL MTT (0.5 mg/mL) was added into 96-well plates and cells were kept to incubate for 4 h. Afterwards, the medium was aspirated off and MTT formazan was dissolved in 200 μL of DMSO. The absorbance at 490 nm was recorded using a microplate spectrophotometer (BioTec, Vermont, VT, USA). Experimental data was expressed as the percentage of the control group.

### 3.3. Immunofluorescence

Immunofluorescence staining of Ki67 and TUNEL were performed to detect cell proliferation and apoptosis, respectively. Cells cultured on coverslips in 35-mm culture dishes were treated with PPD to ensure a discrimination of individual nuclear foci in immunofluorescence staining. After 24 h incubation, cells were fixed with 4% paraform aldehyde for 20 min at room temperature and permeabilized with 0.1% Triton X-100 (Sigma, Louis, MO, USA) for 10 min at 4 °C. After blocking with 5% Bovine Serum Albumin for 30 min at room temperature, cells were stained with TUNEL reagent (Promega, Madison, WI, USA) for 60 min at room temperature in the dark for in situ apoptosis detection; or incubated with antibody against Ki67 (EMD Millipore, Beijing, China) overnight at 4 °C, followed by incubated with Alexa Fluor 488-conjugated goat anti-mouse IgG (Cell Signaling Technology, Danvers, MA, USA) for 1 h at room temperature. Thereafter, samples were counterstained with a mounting medium (Beyotime, Shanghai, China) containing Hoechst 33,342. Three random fields of cell samples were inspected with an Olympus BX61 fluorescence microscope (Olympus, Tokyo, Japan), and images were captured on a Zeiss 510 Meta laser scanning confocal microscope (Zeiss, Jena, Germany). Nuclei containing ≥10 immunoreactive foci were scored as positive for TUNEL.

### 3.4. Flow Cytometry Analysis of Apoptosis and Cell Cycle

To detect cell apoptosis, Hep-2 cells were plated in 6-well plates and treated with different doses of PPD (40 μM, 80 μM and 160 μM), or left untreated. After 24 h, cells were harvested and labeled with Annexin V-FITC-PI Apoptosis Detection Kit (BD Biosciences, San Jose, CA, USA), and then analyzed by flow cytometry (Becton Dickinson, San Jose, CA, USA).

To detect cell cycle, cells were harvested and washed with ice-cold PBS, fixed with ice-cold 70% ethanol at −20 °C for 1 h. After that, cells were briefly washed with 0.2% Triton X-100, subsequently incubated with 100 ng/mL RNase A for 30 min at 37 °C, and then stained with 10 μg/mL PI plus 0.2% Triton X-100 for 30 min at 4 °C in dark. Samples were analyzed by a FACS Canto with FACS Diva Software (Becton Dickinson). The percentage of cells in respective cell cycle phase was calculated using ModFit LT software (ModFit LT for Win32 3.0, Verity Software House, Topsham, Maine, ME, USA).

### 3.5. Western Blotting

Proteins from cells of different treatments were exacted by disruption in RIPA buffer (Sigma-Aldrich, Darmstadt, Germany) in the presence of protease inhibitor cocktail. The lysates were kept on ice for 1 h, and then centrifuged at 12,000× *g* for 20 min at 4 °C. The supernatants were collected for Western blot analysis. Protein concentrations were determined using BCA Protein Quantification Kit (Vazyme biotech, Nanjing, China). Proteins were separated by SDS-PAGE, transferred to PVDF membrane (Merk Millipore, Darmstadt, Germany), and incubated with primary antibodies and secondary antibodies. Blots were developed by enhanced chemiluminescence and images were acquired by Alpha Innotech’s FluorChem imaging system. Primary antibodies used were as follows: mouse anti-human mTOR, eIF4E, 4EBP1, cleaved caspase-3, Bax, Bcl-2 monoclonal antibody (Santa Cruz, Dallas, Texas, USA), CDK1, CDC25 and CCNB1 antibody (Proteintech, Wuhan, China), and rabbit anti-human β-actin. Goat anti-mouse or goat anti-rabbit IgG (Cell Signaling Technology) was used as the secondary antibody.

### 3.6. dSTORM Imaging and Data Analysis

Cells were seeded onto pre-cleaned standard microscope slides and treated with different doses of PPD (40 μM, 80 μM and 160 μM) or left untreated. Then cells were fixed with 4% paraformaldehyde for 10 min at room temperature, permeabilized with 0.1% Triton X-100 for 10 min at 4 °C, and blocked with 5% Bovine Serum Albumin for 30 min at room temperature. After washing by PBS, cells were respectively incubated with anti-human mTOR, eIF4E, and 4EBP1 antibodies overnight at 4 °C, and then stained with Alexa Fluor 647 goat anti-mouse IgG antibodies (Invitrogen, A-21236) for 2 h. At last, cells were incubated with Hoechst 33,342 (1:500 dilution; Sigma) for 10 min. Before imaging, samples were sealed with a STORM imaging buffer containing Tris (50 mM, pH 8.0), NaCl (10 mM), glucose (10% *w*/*v*), β-mercaptoethanol (1% *v*/*v*; Sigma), glucose oxidase (500 μg/mL; Sigma), and catalase (40 μg/mL; Sigma).

dSTORM imaging was performed on a Nikon Ti-E microscope with a 100 × 1.49 NA TIRF lens (Nikon, Japan). In the depth of 2–3 μm above the bottom of the cell, a single image of the nucleus was firstly illuminated by 405 nm laser, and then the Alexa Fluor 647 signals were obtained by 640 nm laser at the same imaging position. All images were captured by a cooled EMCCD camera (Photometrics, Tucson, AZ, USA). Usually, 5000 images were collected for each cell with an internal time of 40 ms between frames to reconstruct a super-resolution image. TetraSpeck microspheres of 100 nm (Invitrogen, Shanghai, China) were embedded in the sample to correct x–y drift, and a focus lock was used to correct z drift. 

Raw dSTORM image sequences were analyzed by QuickPALM [42], a plug-in for Image J processing software (US National Institutes of Health), to obtain a reconstructed dSTORM image. The merging images of the two channels were established by Image-Pro Plus 6.0 (Media Cybernetics, Bethesda, Maryland, MD, USA). To perform image-based cluster analysis, the reconstructed image was converted to a binary image using ’Analysis Particles’ in Image J [43], and the outline of clusters were extracted by setting an appropriate threshold of the area and the circularity of the cluster. Finally, the cluster area and number in one cell were obtained using ‘Measurement’ in Image (Media Cybernetics).

### 3.7. Quantitative Real-Time Reverse Transcription (RT) PCR

Treated cells were lysed in plates after removing the medium. Total RNAs were obtained using Trizol Reagent (Thermo, Waltham, MA, USA). cDNAs were prepared with random hexamers from mRNA, using AMV reverse-transcription kit (Promega). The sequence of primers was as follows: GAPDH: forward 5′-gaaggtgaaggtcggagtc-3′, reverse 5′-gaagatggtgatgggatttc-3′; mTOR: forward 5′-gaggtgtggtttgaccgaag-3′, reverse 5′-atcaggttggatgggtgtct-3′; eIF4E: forward 5′-gttatcagtcccacgcagac-3′, reverse 5′-gaagaggctttggttcagc-3′; 4EBP: forward 5′-ctccttgtgcctccactgat-3′, reverse 5′-gggttcgttcttgtccactt-3′. cDNA amplification reactions were performed using PowerSYBR Green PCR master mix (Thermo). Quantitative PCR reactions were performed on the iQ5™ (BIO-RAD, Hercules, CA, USA). Data were analyzed by a TP800 (Takara, Dalian, China). The 2-ΔΔ*C*_t_ method for relative quantization was used to determine mRNA expression. Fold change was determined as 2-ΔΔ*C*_t_ and target mRNA expression was normalized to GAPDH.

### 3.8. Statistical Analysis

All statistical analysis was performed using SPSS version 19.0 (IBM, Chicago, IL, USA) statistical software package. All data were depicted as mean ± standard deviation (S.D.). Statistical differences between groups were determined by the one-way analysis of variance (ANOVA). Results with *p* < 0.05 were considered statistically significant.

## 4. Conclusions

In summary, by classic biotechnologies and super-resolution fluorescence microscopy, we demonstrate the antitumor effect of PPD on Hep-2 cells and reveal that PPD’s antitumor effect is related to down-regulation of mTOR pathway. PPD invokes strong anticancer activities against Hep-2 cells by inhibiting cell proliferation as well as inducing apoptosis and G2/M cell arrest. Moreover, it down-regulates mTOR signaling pathway by reducing the expression of mTOR and downstream proteins and disrupting the intrinsic clustering distribution of these proteins. These findings form a significant step forward in our understanding of PPD’s antitumor principles. With further studies, a deeper and more comprehensive mapping of PPD targets may be set up and PPD will work as a useful chemotherapeutic agent for the treatment of laryngeal carcinoma.

## Supplementary Materials

Supplementary materials are available online.
