# Supplementary material for: Ginsenoside PPD’s Antitumor Effect via Down-Regulation of mTOR Revealed by Super-Resolution Imaging
Source: Molecules. 2017 Mar 19;22(3):486. doi: 10.3390/molecules22030486 (PMC6155369; doi:10.3390/molecules22030486)
Supplement: Supplementary file 1 [file molecules-22-00486-s001.pdf]

Supplementary Materials

Ginsenoside PPD's antitumor effect via down-regulation of mTOR revealed by super-resolution imaging

Bo Teng, Junguang Jiang, Lijing Zhao, Jing Gao, Junyu Chen, Zhe Liu, Hongda Wang and Binfeng Lu

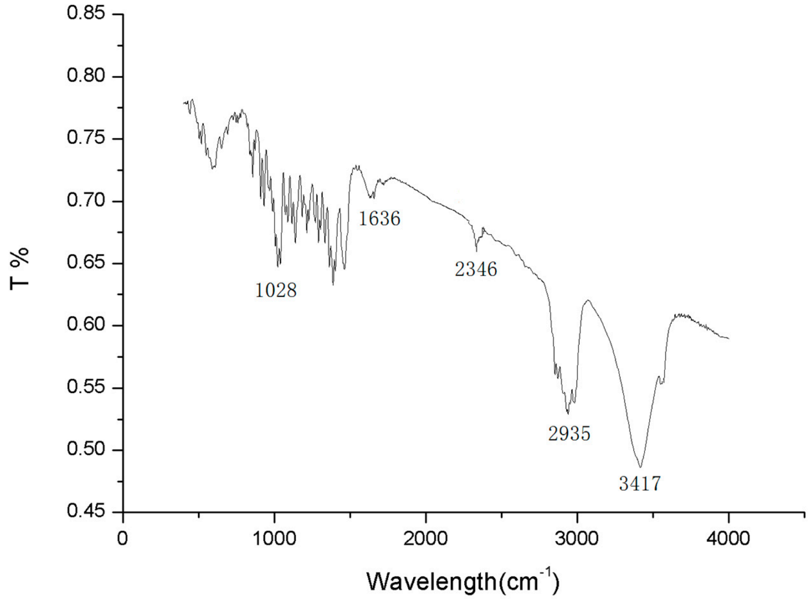

Figure 1. IR of PPD.

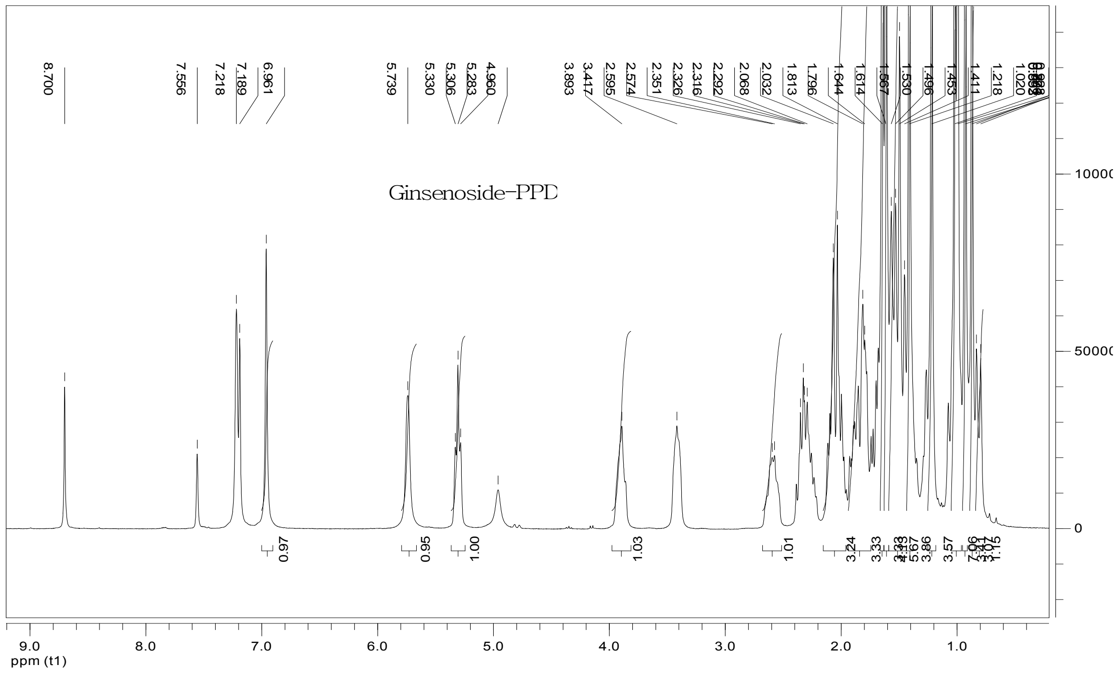

Figure 2. ^{1}H NMR of PPD.

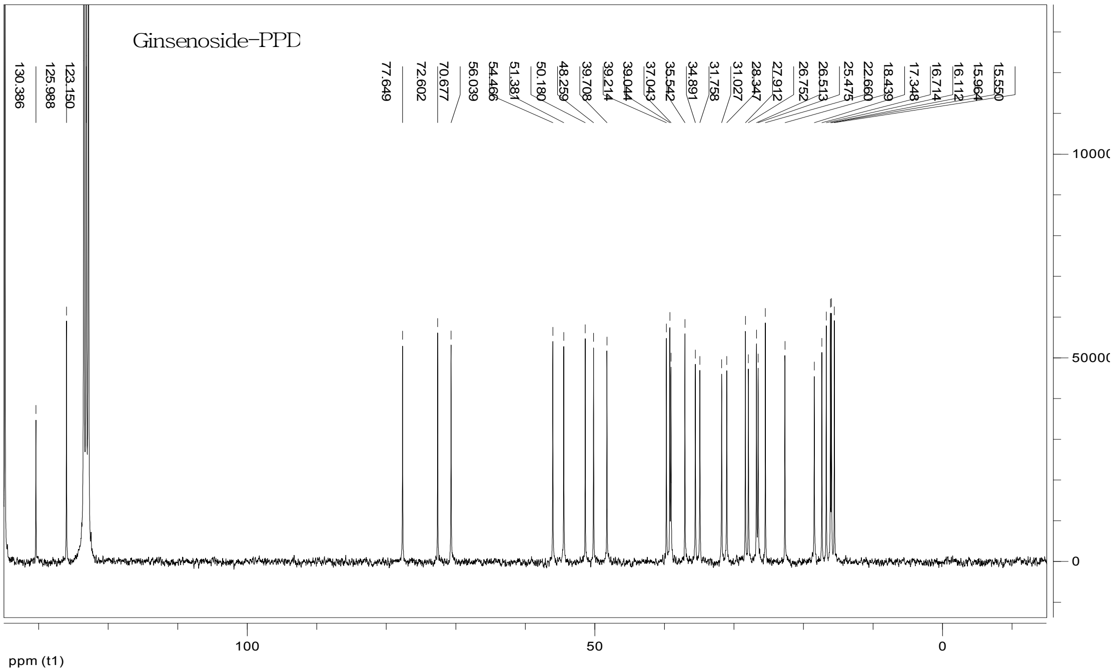

Figure 3. ^{13}C NMR of PPD.
